# Investigation of Tensile Properties and In Situ Analysis of Fracture Behavior in High-Porosity Open-Cell Nickel Foam

**DOI:** 10.3390/ma17215223

**Published:** 2024-10-26

**Authors:** Sufeng Fan, Xihai Wang, Zhe Kong, Qinghua Hou

**Affiliations:** 1 School of Mechanics and Safety Engineering, Zhengzhou University, Zhengzhou 450001, China; 2 Henan Province Engineering Technology Research Center of MEMS Manufacturing and Applications, School of Mechanics and Safety Engineering, Zhengzhou University, Zhengzhou 450001, China; 3 Institute of Intelligent Sensing, Zhengzhou University, Zhengzhou 450001, China

**Keywords:** nickel foam, in situ, tensile, anisotropy, relative density, fracture process

## Abstract

Nickel foam offers excellent conductivity, a high surface area, and lightweight structure, making it ideal for applications, like battery electrodes, catalysts, and filtration systems. Its durability and corrosion resistance further enhance its performance in various industries. However, few studies focus on the tensile anisotropy of nickel foam and its tensile fracture process. In this study, the anisotropic tensile behavior of nickel foam with varying relative densities has been investigated, along with its tensile fracture behavior using in situ techniques. The tensile properties of nickel foams show strong anisotropy due to the flattening process in the production process. The results show that the tensile properties, including the yield strength, tensile strength, and elastic modulus, increase with the increasing relative density, while the elongation percentage has no relationship with the relative density. The experiment data on tensile strength are in agreement with Gibson’s formula and Liu’s formula. In situ tensile tests are conducted to explore the microscopic fracture mechanism of nickel foam. The results show that the struts of nickel foam are tensile fractures or shear fractures near the joints, and the fracture process of struts is clearly recorded and analyzed. This study is significant as it provides critical insights into the anisotropic tensile behavior of nickel foam and fracture mechanism, enabling the optimization of production processes and broadening its potential applications.

## 1. Introduction

Metal foams are highly attractive for a wide range of industrial applications due to their exceptional mechanical and functional properties, including high specific strength, excellent energy and sound absorption, high flame resistance, and superior vibration dampening [1,2]. They are generally classified into two types, closed-cell and open-cell foams, based on whether the cells within the foam are interconnected. Closed-cell foams are particularly notable for their extended stress plateau during compressive deformation, making them ideal for cushioning impact and absorbing acoustic energy, often used in packaging [3,4,5,6,7]. Open-cell metal foams with high porosity are also particularly significant among porous materials. The three-dimensional structure and large specific surface area result in key features, such as high permeability, excellent damping capabilities, a high surface-to-volume ratio, and exceptional electrical and thermal conductivity. These characteristics make them well suited for applications, like thermal management in electronics, battery electrodes, catalyst supports, exhaust gas recirculation filters, and lightweight structural components [4,8,9,10,11,12]. The increasing demand for portable power sources in personal electronics has propelled the battery market to search for compact and long-lasting batteries, and the structure of the electrode plays a crucial role in enhancing battery performance [13,14].

Open-cell nickel foam, with a porosity exceeding 95% by volume, is one of the most widely used metal foams, particularly in nickel–cadmium (Ni-Cd) and nickel–metal hydride (Ni-MH) batteries [15,16]. Its exceptionally high porosity gives it unique properties and applications, setting it apart from other metal foams [17]. It is commonly employed as an electrode material in rechargeable batteries, being infiltrated with an active paste of electrochemical material like nickel oxide [18,19]. The volume of this active paste changes various cycles, including charging, discharging, recharging, and overcharging. If the tensile strength is inadequate, the foam may crack, either during production or in use. In general, nickel foam is often exposed to mechanical loading, making high strength a critical factor. Therefore, understanding and analyzing their mechanical behavior are essential [10,20,21,22,23].

Despite its significance, research on these mechanical aspects has been rather limited. Most studies on the mechanical properties of metal foam have focused on closed-cell foam metals such as aluminum foam and have mainly investigated their compressive properties [24,25,26,27]. Consequently, there are even fewer studies on the mechanical properties of nickel foam, especially the tensile properties. Fan et al. studied the compressive properties of nickel foams, including yield strength, elastic modulus, energy absorption density, and energy absorption efficiency [28]. The findings revealed that yield strength, elastic modulus, and energy absorption density increase with the increasing relative density of the foams. Feshat et al. [29] investigated the mechanical behavior of nickel foams for potential use in lithium battery applications, using polyurethane foam as a precursor and electrodeposition for production, along with simulation methods. Mohammad Shaheta [20] reported a tensile strength of approximately 0.65 MPa for nickel foams. Additionally, Levy et al. [30] produced open-cell nickel foams and explored the impact of carbon coatings on their properties, which are commonly used in supercapacitors. However, few studies have specifically investigated the tensile properties and fracture behavior of nickel foam.

In this study, the relationship between tensile properties and relative density is studied, as well as the anisotropy of tensile properties in nickel foam. The fracture process of nickel foam during the tensile process is studied through in situ optical microscopy (OM) and in situ scanning electron microscope (SEM) techniques.

## 2. Materials and Methods

Open-cell nickel foams with different relative densities were purchased from Changde Liyuan New Materials Co., Ltd (Changde, China). The tensile samples were cut into strips, 150 mm long, 20 mm wide, and 1.8 mm thick, and the gauge length was 100 mm. The specimens are not dumbbell-shaped because some defects may occur on the specimens during the process of sample preparation. The tensile tests are performed on a SUST CMT-1104 universal testing machine (Zhuhai, China) at ambient temperature.

In the relative density experiment, five groups of specimens with surface area densities of 250 g/m^3^, 280 g/m^3^, 320 g/m^3^, 350 g/m^3^, and 420 g/m^3^ are used. The relative density of nickel foams is defined as the ratio of the mass density of the nickel foam to that of the pure nickel. Thus, the density of the nickel foam can be expressed as the following equation:

(1) $$\rho *=\frac{\rho_{foam}}{\rho_{s}}$$

where ρ_foam_ is the surface area density of the porous material. ρ_s_ stands for the density of the corresponding compact material, and it is 8.908 g/cm^3^. ρ^*^ is the relative density. The relative density of the five groups’ sample is 1.56%, 1.75%, 1.995%, 2.18% and 2.62%, respectively. The specimens are stretched at a constant strain rate of 1.67 × 10^−3^s^−1^, and the mean values without abnormal data are taken for the final experiment data. Moreover, only the data where the fracture occurs in the gauge area of the sample will be considered valid. The calculation method of stress is to divide the force data by the cross-section of the nickel foam tensile sample.

The in situ OM (Micromanipulator 4060 Manual Probe Station, Carson City, NV, USA) and SEM (FEI Quanta^TM^ 450 FESEM, Hillsboro, OR, USA) tensile tests are performed with Gatan^TM^ Microtest (Pleasanton, CA, USA). The nickel foam samples with a relative density of 1.56% are cut into strips suitable for clamping. The tensile strain rate is 10^−3^s^−1^. The tensile process is captured by OM and SEM.

During the production process of nickel foams, the samples are slightly stretched to ensure the smoothness of the nickel foams. This stretching can result in anisotropy. Therefore, tensile experiments are carried out in two directions to investigate the anisotropy of nickel foams. The morphology of the nickel foam is shown in Figure S1 in the *Supplementary Information*. The pore size ranges from ~70 μm to ~400 μm. In this study, the tensile direction is referred to as the longitudinal direction (LD), while the direction perpendicular to the tensile direction is called the transverse direction (TD).

## 3. Results and Discussion

### 3.1. Influence of Relative Density on Tensile Properties of Nickel Foam

**Figure 1:**
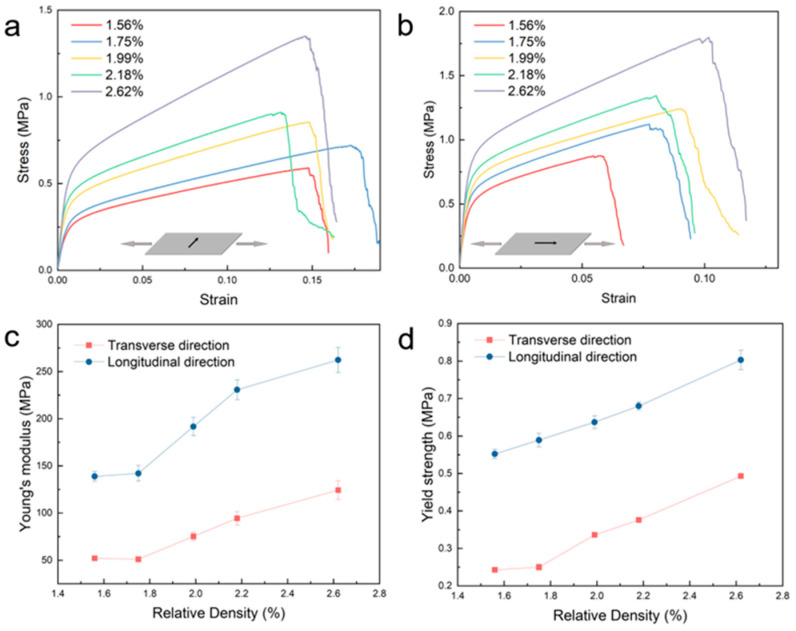
Relationship between mechanical properties and relative density of nickel foam. (**a**) The tensile stress–strain curve of nickel foam in the transverse direction. (**b**) The tensile stress–strain curve of nickel foam in the longitudinal direction. (**c**) The change in Young’s modulus with relative density in both directions. (**d**) The change in yield strength with relative density in both directions.

Figure 1a,b show the stress–strain curve of nickel foams with relative densities of 1.56%, 1.75%, 1.99%, 2.18%, and 2.62% in the longitudinal direction and transverse direction. In both directions, Young’s modulus and tensile strength increase with the increase in relative density. As shown in Figure 1c, the Young’s modulus increases from 52 MPa to 124 MPa in the transverse direction, with the relative density increasing from 1.56% to 2.62%. In the longitudinal direction, Young’s modulus increases from 139 MPa to 262MPa, with the relative density increasing from 1.56% to 2.62%. The tensile yield strength follows the same rule. As shown in Figure 1d, in the transverse direction, the yield strength increased from 0.24 MPa to 0.493 MPa with relative density increases from 1.56% to 2.62%. In the longitudinal direction, the yield strength increases from 0.55 MPa to 0.80 MPa, with relative density increases from 1.56% to 2.62%. In contrast, the elongation does not show a clear correlation with relative density, as shown in Figure 1a,b.

Figure 1 shows the tensile strength, yield strength, and elastic modulus increase with the increasing relative density in both directions. The reason for this phenomenon is that the thickness of the metal nickel layer of the nickel foam with different relative densities is different. The production process of nickel foam is first to electroplate a metal nickel layer on top of the polyurethane foam, then burn off the polyurethane foam, leaving behind the metal nickel layer skeleton. Next, it is reduced with hydrogen and finally undergoes heat treatment. The production process of nickel foams reveals that the structure of nickel foam is similar to that of the original polyurethane foams. Thus, the difference among nickel foams with different relative densities lies in the thickness of the nickel layer deposited on the original foams. A sample with a higher relative density has a thicker nickel layer and, therefore, exhibits better mechanical properties. The insert in Figure S1 in the *Supplementary Information* shows the cross-section of a strut of nickel foam.

Gibson et al. proposed a formula to indicate the relative density and the tensile strength, as shown in Formula (2) [31,32,33]. σ is the plastic collapse stress of the porous material. σ_ys_ is the yield stress of the full dense material. ρ_foam_ is the surface area density of the porous material, and ρ_s_ stands for the density of the corresponding compact material. $\frac{\rho_{foam}}{\rho_{s}}$ is the relative density of the porous material. C_1_ is a constant, which is decided by the experiment. Substituting the experiment data and σ_ys_ = 148 MPa [34] into Formula (2) can elicit the mean value of constant C. Because of the anisotropy of the tensile properties, data in different directions reach different constants. For the transverse direction, the constant C_TD_ is 293.6, while for the longitudinal direction, the constant C_LD_ is 451.7.

(2) $$\sigma \approx C*{\left(\frac{\rho_{foam}}{\rho_{s}}\right)}^{3/2}*\sigma_{ys}$$

Liu et al. [35,36] also proposed a formula to indicate the relative density and the tensile strength, as shown in Formula (3). σ is the tensile strength of the porous material. σ_0_ stands for the tensile strength of the dense material, and K is a constant. In the same way, substituting the experimental data and σ_0_ = 462 MPa [37] into Equation (3) can show the mean value of the constant K. For the transverse direction, the constant K_TD_ is 110.4, while for the longitudinal direction, the constant K_LD_ is 169.1.

(3) $$\sigma \approx K*{\left(\frac{\rho_{foam}}{\rho_{s}}\right)}^{1.25}*\sigma_{0}$$

**Figure 2:**
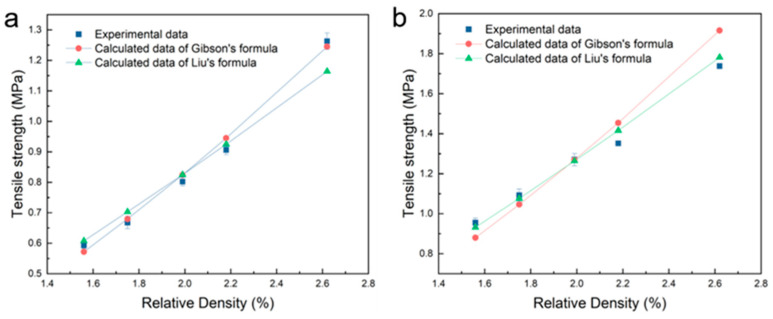
The comparison between the experimental data of tensile strength and the calculation data of Gibson’s formula and Liu’s formula in transverse direction (**a**) and longitudinal direction (**b**).

Figure 2a presents a comparison between the experimental data of tensile strength in the transverse direction and the calculation data of two formulas (raw data are shown in Tables S1 and S2 in *Supplementary Information*). The results show that for the samples with relative densities ranging from 1.56% to 2.18%, the experimental results are in good agreement with the calculated results of the two formulas. However, for the sample with a relative density of 2.62%, the experimental results are obviously in good agreement with the calculation results of Gibson’s formula, but there is a certain gap between them and the calculation results of Liu’s formula. Figure 2b shows a comparison between the experimental data of tensile strength in the longitudinal direction and the calculation data of two formulas. For the samples with relative densities from 1.56% to 1.99%, the experimental data agree well with the results calculated by Liu’s formula but are somewhat different from those calculated by Gibson’s formula. For the samples with a relative density from 2.18% to 2.18%, the experimental results are slightly different from the results calculated by both formulas, but the difference from the results calculated by Liu’s formula is smaller. Therefore, in general, the experimental results in the transverse direction are more consistent with Gibson’s formula, while the experimental results in the longitudinal direction are more consistent with Liu’s formula.

### 3.2. Anisotropy of Nickel Foams’ Tensile Properties

**Figure 3:**
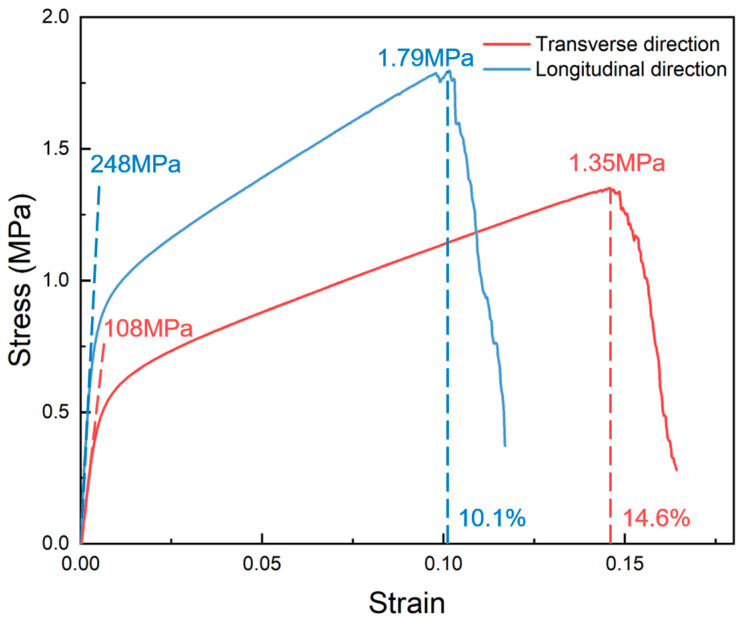
The tensile stress–strain curves of nickel foam with a relative density of 2.62% in two directions.

Figure 1 and Figure 2 indicate that the tensile properties of nickel foam have obvious anisotropy. Figure 3 shows the tensile stress–strain curves of nickel foam with a relative density of 2.62% in two directions. Young’s modulus exhibits significant anisotropy, with a value of 108 MPa in the transverse direction, in contrast to the considerably higher value of 248 MPa in the longitudinal direction. The tensile strength in the transverse direction is 1.35 MPa, while the value in the longitudinal direction is 1.79 MPa. The yield strength in the transverse direction is 0.44 MPa, while the value in the longitudinal direction is 0.75 MPa. However, the transverse elongation is better than the longitudinal elongation, 14.6% and 10.1%, respectively.

The source of anisotropy is the flattening process in the production process of nickel foam. In the production of nickel foam, once the metal nickel layer is plated onto the surface of the polyurethane foam, the polyurethane foam is incinerated, leaving only the metal nickel skeleton. Subsequently, a reduction process using hydrogen and a heat treatment process are carried out [38]. However, these processes can lead to an overall uneven phenomenon in the nickel foam. To make the surface of the nickel foam flatter, a certain degree of stretching is applied to the nickel foam. Therefore, the structure of the nickel foam is elongated in the longitudinal direction compared to the transverse direction. In addition to affecting the structure of nickel foam, the stretching process in the production process also causes work hardening in the longitudinal direction of nickel foam. Therefore, the result is that the Young’s modulus, yield strength, and tensile strength of nickel foam in the longitudinal direction are higher than that in the transverse direction, but the elongation in the longitudinal direction is worse than that in the transverse direction.

### 3.3. Fracture Process Analysis Through In Situ OM and SEM

**Figure 4:**
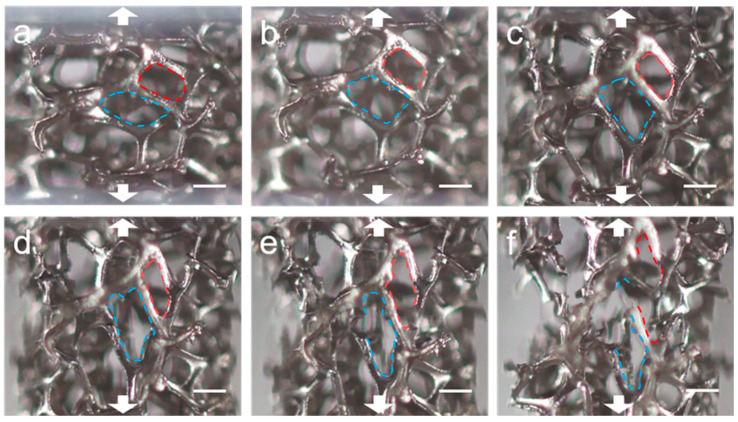
The deformation process of two nickel foam cells during the tensile process. (**a**–**c**) The shape changes of nickel fam cells in the initial stage of stretching. (**d**–**f**) As the stretching progresses, fractures begin to occur. The red line and the blue line are used to analyze the fracture processes of two cells frame by frame. Scale bar: 200 μm.

In situ OM tests are conducted to investigate the fracture process during the tensile process of nickel foam (*Supplementary Video S1*). Figure 4 shows the deformation process of two nickel foam cells during the tensile process. From Figure 4a–d, the red cell is gradually deformed along the stretching direction. As shown in Figure 4e, the strut at the bottom-right side of the red cell was pulled off. The break does not occur in the middle of the strut but near the joint. At the same time, a crack appears on the right side near the joint and eventually breaks completely in Figure 4f. The blue cell is in a similar situation. From Figure 4a–c, the shape of the blue cell, which is somewhat flat, is gradually elongated along the stretching direction during the stretching process, and the shape changes dramatically. In Figure 4d, the strut on the upper-left side of the blue cell cracks and eventually breaks near the joint in Figure 4e. At the same time, the strut on the upper left of the blue cell has a crack. This strut is also the strut on the left side of the red cell and eventually breaks near the joint in Figure 4f. From the analysis of the fracture process of the red and blue nickel foam cell in Figure 4 during the tensile process, the struts often break near the joints, rather than in the middle. The in situ SEM technique is needed to investigate why the struts break near the joints.

**Figure 5:**
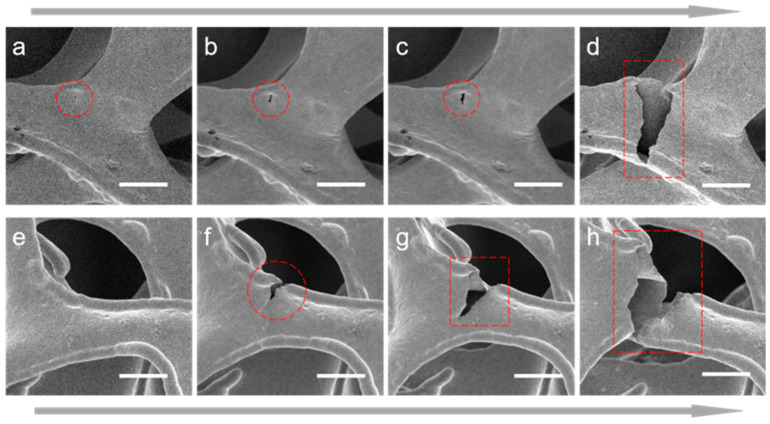
Crack propagation during the in situ tensile process of nickel foam. (**a**–**d**) Crack began from the defects on the surface, and the process of crack propagation until fracture. (**e**–**h**) Crack began from the edge of the strut, and the process of crack propagation until fracture. Scale bar: 50 μm.

Figure 5 shows the fracture process of two struts during the tensile process. The in situ SEM technique is employed to analyze the fracture process. One cause of fractures is that the defects on the surface of nickel foam develop into cracks under the action of tensile stress and eventually lead to fracture. As shown in Figure 5a, under the action of tensile stress, the surface defects (in the red circle) at the joint of nickel foam generate cracks due to stress concentration. From Figure 5b,c, the crack gradually expands, and the fracture occurs in Figure 5d (*Supplementary Video S2*). Another cause of fracture is that at the edges of nickel foam, due to stress concentration, cracks develop, leading to the fracture of the struts. As shown in Figure 5e, due to production process problems, the surface of the nickel foam edge is not smooth and uniform. As a result, when the overall structure is subjected to tensile stress, stress concentration will occur somewhere at the edge, resulting in cracks, as shown in Figure 5f. As shown in Figure 5g,h, the crack gradually expands under the action of tensile stress, resulting in fracture (*Supplementary Video S3*).

### 3.4. Discussion

This study focuses on the influence of relative density and anisotropy on the tensile properties of nickel foam. At the same time, in situ tensile technology is used to study the fracture process of nickel foam at the microscopic level. Firstly, according to the production process of nickel foam, the difference between nickel foams with different relative densities lies in the different thicknesses of the metal nickel layer. Therefore, the mechanical properties of nickel foam, such as yield strength, tensile strength, and Young’s modulus, increase with an increase in relative density. However, since nickel foam has a porosity of more than 95%, its elongation is mainly affected by its structure. Therefore, the tensile elongation does not show a correlation with the relative density. The tensile strength experiment data are in excellent accordance with Gibson’s formula and Liu’s formula. Secondly, in the production process of nickel foam, in order to make the surface of nickel foam flat, a certain degree of stretching is carried out at both ends, which will cause anisotropy in the nickel foam. In previous studies on the tensile properties of nickel foam, the influence of anisotropy on tensile properties has not been studied [20,39,40,41]. This study shows that the yield strength, tensile strength, and Young’s modulus of nickel foam in the longitudinal direction are stronger than those in the transverse direction, while the elongation rate is the opposite. Thirdly, previous studies often focused on the macroscopic fracture process of nickel foam while ignoring the microscopic fracture behavior [20,39,40,41]. In this study, in situ tensile technology is used to study the fracture process of nickel foam at the microscopic scale, and frame-by-frame analysis is carried out. The results show that nickel foam often fractures near the joint. Cracks nucleate from the surface or edge. Pores or defects on the surface may lead to the appearance of cracks.

## 4. Conclusions

In this study, the tensile properties of nickel foam with different relative densities are studied, and the tensile fracture process is investigated using in situ OM and SEM techniques for the first time. The experimental results show that with an increase in relative density (from 1.56% to 2.62%), the tensile properties, such as tensile strength, yield strength, and elastic modulus, of the material are all improved, but the elongation does not show a correlation with the relative density. Due to the flattening process in the production of nickel foam, the tensile properties of nickel foam show obvious anisotropy. Young’s modulus, yield strength, and tensile strength in the longitudinal direction are higher than those in the transverse direction. However, the elongation shows an opposite trend. The in situ OM and SEM techniques are used to investigate the tensile fracture process of nickel foam. The results show that the nickel foam often breaks at the joint, and the defects on the surface of the sample or the stress concentration at the edge of the strut will lead to cracks, resulting in fracture. This study provides reliable data support for the application of nickel foam in different fields, such as the battery, filtration, and catalysis fields, and can help manufacturers improve the product design and production process, improving the product performance and quality.

## Data Availability

The original contributions presented in this study are included in the article/Supplementary Material; further inquiries can be directed to the corresponding author/s.

## Supplementary Materials

The following supporting information can be downloaded at: https://www.mdpi.com/article/10.3390/ma17215223/s1, Figure S1: The structure of nickel foam; Table S1: Tensile strength data in the transverse direction of the nickel foams and relative calculation values of the formulas; Table S2: Tensile strength data in the longitudinal direction of the nickel foams and relative calculation values of the formulas; Video S1: the in situ OM tensile test video shown in Figure 4; Video S2: the in situ SEM tensile test video shown in Figure 5a–d; Video S3: the in situ SEM tensile test video shown in Figure 5e–h.
