# Supplementary material for: Investigation of Tensile Properties and In Situ Analysis of Fracture Behavior in High-Porosity Open-Cell Nickel Foam
Source: Materials (Basel). 2024 Oct 26;17(21):5223. doi: 10.3390/ma17215223 (PMC11547180; doi:10.3390/ma17215223)
Supplement: Supplementary file 1 [file materials-17-05223-s001.zip › Supplementary Figure and Tables.pdf]

Supplementary information for

Investigation of Tensile Properties and In Situ Analysis of Fracture Behavior in High-Porosity Open-Cell Nickel Foam

Sufeng Fan [1,2,3,*], Xihai Wang [1,2,3], Zhe Kong [1,2,3] and Qinghua Hou [1,2,3]

1 School of Mechanics and Safety Engineering, Zhengzhou University, Zhengzhou 450001, China

2 Henan Province Engineering Technology Research Center of MEMS Manufacturing and Applications, School of Mechanics and Safety Engineering, Zhengzhou University, Zhengzhou 450001, China

3 Institute of Intelligent Sensing, Zhengzhou University, Zhengzhou 450001, China

* Correspondence: fansufeng@zzu.edu.cn

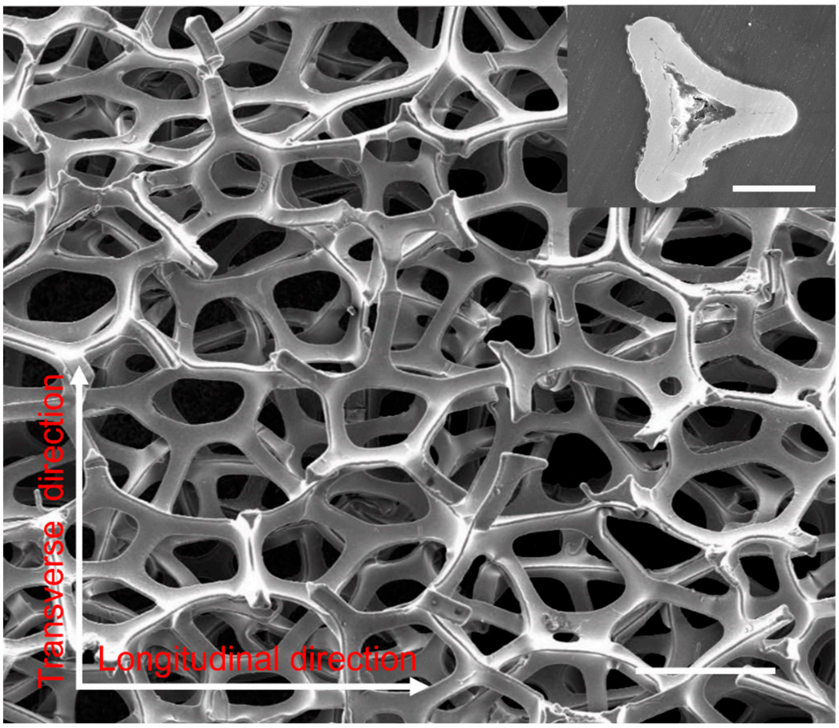

Figure S1. The structure of nickel foam. Scale bar: 500μm. Insert: the cross section of a strut of nickel foam. Scale bar: 40μm.

Table S1. Tensile strength data in the transverse direction of the nickel foams and relative calculation values of the formulas.

Number	1	2	3	4	5
Relative density/%	1.56	1.75	1.99	2.18	2.62
σ′(MPa)	0.593	0.681	0.802	0.907	1.286
σ-F(2)(MPa)	0.572	0.680	0.824	0.945	1.245
σ-F(3)/(MPa)	0.608	0.703	0.825	0.925	1.164
abs(Δσ/σ′) -F(2)(%)	3.5	0.1	2.7	2.0	3.2
abs(Δσ/σ′) -F(3)(%)	2.5	3.2	2.9	2.0	9.5

Where: σ′: experiment value of the tensile strength; σ: calculation value of tensile strength; Δσ=σ-σ′.

Table S2. Tensile strength data in the longitudinal direction of the nickel foams and relative calculation values of the formulas.

Number	1	2	3	4	5
Relative density/%	1.56	1.75	1.99	2.18	2.62
σ'(MPa)	0.975	1.105	1.270	1.352	1.738
σ-F(2)(MPa)	0.880	1.046	1.268	1.454	1.916
σ-F(3)(MPa)	0.932	1.076	1.264	1.416	1.782
abs($\Delta\sigma/\sigma'$) -F(2)(%)	9.7	5.3	0.2	7.5	10.2
abs($\Delta\sigma/\sigma'$) -F(3)(%)	4.4	2.6	0.5	4.7	2.5

Where the symbols mean the same as that in Table S1..
